# The Time Sequence of Face Spatial Frequency Differs During Working Memory Encoding and Retrieval Stages

**DOI:** 10.3389/fpsyg.2022.853992

**Published:** 2022-05-20

**Authors:** Anqing Wang, Enguang Chen, Hang Zhang, Chinheg H. Borjigin, Hailing Wang

**Affiliations:** School of Psychology, Shandong Normal University, Jinan, China

**Keywords:** face, configural processing, spatial frequency, working memory, coarse-to-fine sequence

## Abstract

Previous studies have found that P1 and P2 components were more sensitive to configural and featural face processing, respectively, when attentional resources were sufficient, suggesting that face processing follows a coarse-to-fine sequence. However, the role of working memory (WM) load in the time course of configural and featural face processing is poorly understood, especially whether it differs during encoding and retrieval stages. This study employed a delayed recognition task with varying WM load and face spatial frequency (SF). Our behavioral and ERP results showed that WM load modulated face SF processing. Specifically, for the encoding stage, P1 and P2 were more sensitive to broadband SF (BSF) faces, while N170 was more sensitive to low SF (LSF) and BSF faces. For the retrieval stage, P1 on the right hemisphere was more sensitive to BSF faces relative to HSF faces, N170 was more sensitive to LSF faces than HSF faces, especially under the load 1 condition, while P2 was more sensitive to high SF (HSF) faces than HSF faces, especially under load 3 condition. These results indicate that faces are perceived less finely during the encoding stage, whereas face perception follows a coarse-to-fine sequence during the retrieval stage, which is influenced by WM load. The coarse and fine information were processed especially under the low and high load conditions, respectively.

## Introduction

Visual stimuli perception combines multiple hierarchical levels of information. According to numerous studies, these different levels are processed at different times and follow a coarse-to-fine sequence (Boeschoten et al., 2005; Hegde, 2008; Goffaux et al., 2011; Petras et al., 2019). The global-local letter paradigm (Navon stimuli) has been widely used to investigate how these levels are processed. Results from these investigations suggest that global processing precedes the analysis of local information (Perfect et al., 2008; Gao et al., 2011).

As a complex visual stimulus, a face contains configural and featural information. Configural face information included first-order relational (i.e., the arrangement of face features with two eyes above a nose, which is above a mouth), second-order relational (i.e., the distance between facial features, such as the distance between eyes or the distance between the mouth and nose), and holistic processing (i.e., the gestalt of face features), whereas featural face information referred to the differences in face internal features, such as the shape or size of eyes and mouth (Maurer et al., 2002). Electrophysiological studies have importantly contributed to our understanding of the time course of configural and featural face processing in the human brain. Event-related potential (ERP) studies showed that configural and featural processing elicit separate responses as early as 100–250 ms after the onset of face presentation. For example, Wang et al. (2016) reported that second-order relational and featural face information elicited a larger P1 (approximately 128 ms) and P2 (approximately 248 ms), respectively, under the face-attended condition. These results, which were later replicated by several studies (Wang and Fu, 2018; Wang et al., 2020), suggest the temporal precedence of second-order relational over featural face processing and lend support to coarse-to-fine face processing. However, in the featural face sets utilized in the above-mentioned studies, the eyes or mouth in a given face were replaced by the eyes or mouth from a separate face and the feature location did not change. As Leder and Carbon (2006) pointed out, this kind of featural face manipulation requires second-order relational processing as the second-order relationships are also inherently changed. This subtle but important difference between second-order relational and featural manipulation might not have been purely dissociated in previous studies.

Notably, any early visual input contains different spatial frequencies based on the spatial frequency theory (Shulman and Wilson, 1987; Morrison and Schyns, 2001; Jeantet et al., 2018). Many previous studies have demonstrated an association between spatial frequency and global/local processing. Specifically, local details (i.e., fine information) of an image are represented by high spatial frequency (HSF) information, whereas large-scale visual details (i.e., coarse information) are represented by low spatial frequency (LSF) information (Rolls et al., 1985; Nagayama et al., 1995). Thus, previous studies have proposed that local (fine) and global (coarse) information are transmitted by relatively high and low spatial frequencies, respectively (Evans et al., 2000; Malinowski et al., 2002; Flevaris et al., 2009). Similarly, other studies investigating face-specific SF processing found that LSF and HSF information played a dominant role in face configural and featural processing, respectively (Boeschoten et al., 2005; Goffaux et al., 2005; Jeantet et al., 2019). For example, Goffaux et al. (2005) altered face second-order relational and featural information for both LSF and HSF conditions. They reported better performance during the LSF condition after changing face second-order relational information and better performance during the HSF condition after altering face featural information, suggesting that processing of second-order relational and featural information of faces can be dissociated by face spatial frequency. Thus, it is believed that configural face processing requires LSF information and featural face processing requires HSF.

It has been shown that the processing of LSF faces precedes that of HSF faces (Wang et al., 1998; Gao and Bentin, 2011; Peters et al., 2018), which supports the coarse-to-fine theory of facial processing. An fMRI study showed that most face-sensitive brain regions robustly responded to LSF faces during the early stage (75 ms) but exhibited decreased activation from 75 ms to 150 ms and, conversely, showed heightened activation during processing of HSF faces from 150 to 300 ms (Goffaux et al., 2011). Moreover, Jeantet et al. (2019) asked participants to perform a facial gender discrimination task on LSF and HSF faces and observed that LSF and HSF faces elicited a larger P1 and N170 (about 160 ms) amplitude, respectively. These results were in line with the coarse-to-fine theory of face perception, but not all studies have yielded congruent results (Goffaux et al., 2003; Mares et al., 2018). In the study by Goffaux et al. (2003), which employed a training-test paradigm, LSF faces elicited a larger N170 amplitude relative to HSF faces in the gender task but not in the familiarity task, and no difference in P1 elicited by LSF and HSF faces was observed. Taken together, these studies suggest that the time course of face spatial frequency processing is best examined with studies of ERP components. However, there is less evidence supporting this claim for the late-stage components, such as P2, which is linked to deeper processing and increasing sensory demand (Mercure et al., 2008). And there was a conflict about the P2 results. Some studies found that it was more sensitive to featural face information (Wang et al., 2016; Wang et al., 2018), while others found that it was more sensitive to second-order relational information (Mercure et al., 2008).

Furthermore, the coarse-to-fine face processing occurred when attentional resources were sufficient (Wang et al., 2016; Jeantet et al., 2019) as only one face stimulus was presented. It is unclear whether the time course of LSF and HSF face processing was impacted by the resource limitations, which can be characterized by working memory capacity. Previous studies demonstrated that working memory (WM) capacity for objects can vary based on qualitative differences in configural and featural processing. WM capacity can be measured by experimentally manipulating WM load, which is defined as the number of items maintained simultaneously (Morgan et al., 2008; Bauser et al., 2011). Generally, upright face stimuli are perceived by configural processing to a greater degree than inverted faces or other objects (Maurer et al., 2002). A behavioral study found that participants had a larger WM capacity for upright faces than for inverted faces and upright cars at a longer encoding duration, suggesting that configural processing might contribute to the increasing WM capacity when sufficient time is given for encoding (Curby and Gauthier, 2007). However, another behavioral study reported discrepant results that face configural processing was impaired under a high WM load condition relative to a WM load condition (Cheung and Gauthier, 2010).

Furthermore, within a delayed recognition task paradigm, previous studies found that WM load effects on the processing of upright faces and human body forms, which are perceived in a configural-based manner, are dissociated during the WM encoding and retrieval stages on early ERP components (Morgan et al., 2008; Bauser et al., 2011). Specifically, P1 (from load 1 to 2) and N170, which were evoked by upright faces, increased with increasing WM load during the encoding stage, whereas both P1 and N170 elicited by human body forms were not influenced by WM load at the encoding stage. But for the retrieval stage, both studies found that N170 decreased with increasing WM load and that P1 showed no differences across WM load. In addition, P3b (~300–700 ms), which is strongly associated with WM and an attentional updating process (Polich, 2007), was suppressed by increasing WM load during the encoding and retrieval stages. In addition, Morgan et al. (2008) analyzed the N250r component, which is more negative for familiar faces than unfamiliar faces, and it is assumed that it reflects WM processes related to temporary activation of face recognition units. They found that the amplitude of N250r decreased as WM load increased at both encoding and retrieval stages. Taken together, these studies demonstrated that the neural mechanisms underlying the processing of these objects, which occur configurally, differ during WM encoding and retrieval periods, especially on P1 and N170 components. However, few studies focused on featural processing mechanisms during WM encoding and retrieval periods. Importantly, evidence supporting coarse-to-fine sequential processing during WM encoding and retrieval stages is currently lacking.

To this end, this study used a delayed recognition task to explore whether and how the WM load influences the time sequence of configural and featural face processing during WM encoding and retrieval stages. We examined configural and featural face processing by means of spatial frequency filtering. Stimuli were either unfiltered and contained all spatial frequencies (BSF), low-pass filtered (LSF), or high-pass filtered (HSF). Based on the previous ERP studies investigating configural and featural face processing (Mercure et al., 2008; Lv et al., 2015; Negrini et al., 2017; Wang et al., 2020), we analyzed the P1, N170, and P2 components to investigate the time course of LSF and HSF face processing during encoding and retrieval stages, and we analyzed P3b and N250r components to test the effect of WM load. We hypothesize that LSF processing will precede HSF processing at both memory encoding and retrieval stages if face processing occurs in a coarse-to-fine manner during both the working memory processing stages. If memory load impacts the time course of featural and configural face processing, then the ERP components elicited by faces with differing spatial frequencies would differ as a function of WM load. According to Wang and Fu (2018), coarse-to-fine processing occurs when attentional resources are sufficient; thus, in this study, we speculate that coarse-to-fine sequential processing will be found under low, but not high, WM load conditions.

## Materials and Methods

### Participants

A power analysis using G^*^Power software version 3.01 (Faul et al., 2007) indicated that for an effect size of 0.2, at least 22 participants were required to achieve 80% power. In this study, 29 students (23 female students, aged 19.34 ± 1.21 years) completed the experiment, but one was excluded from the behavioral performance due to a data recording error. All participants were right-handed based on Edinburgh Handedness Test. All participants had normal or corrected-to-normal vision without color blindness or mental illness based on self-report. All study procedures were approved by the local Institutional Review Board of the School of Psychology, Shandong Normal University. Written informed consent was collected from all participants prior to the study initiation.

### Stimuli and Apparatus

**Figure 1:**
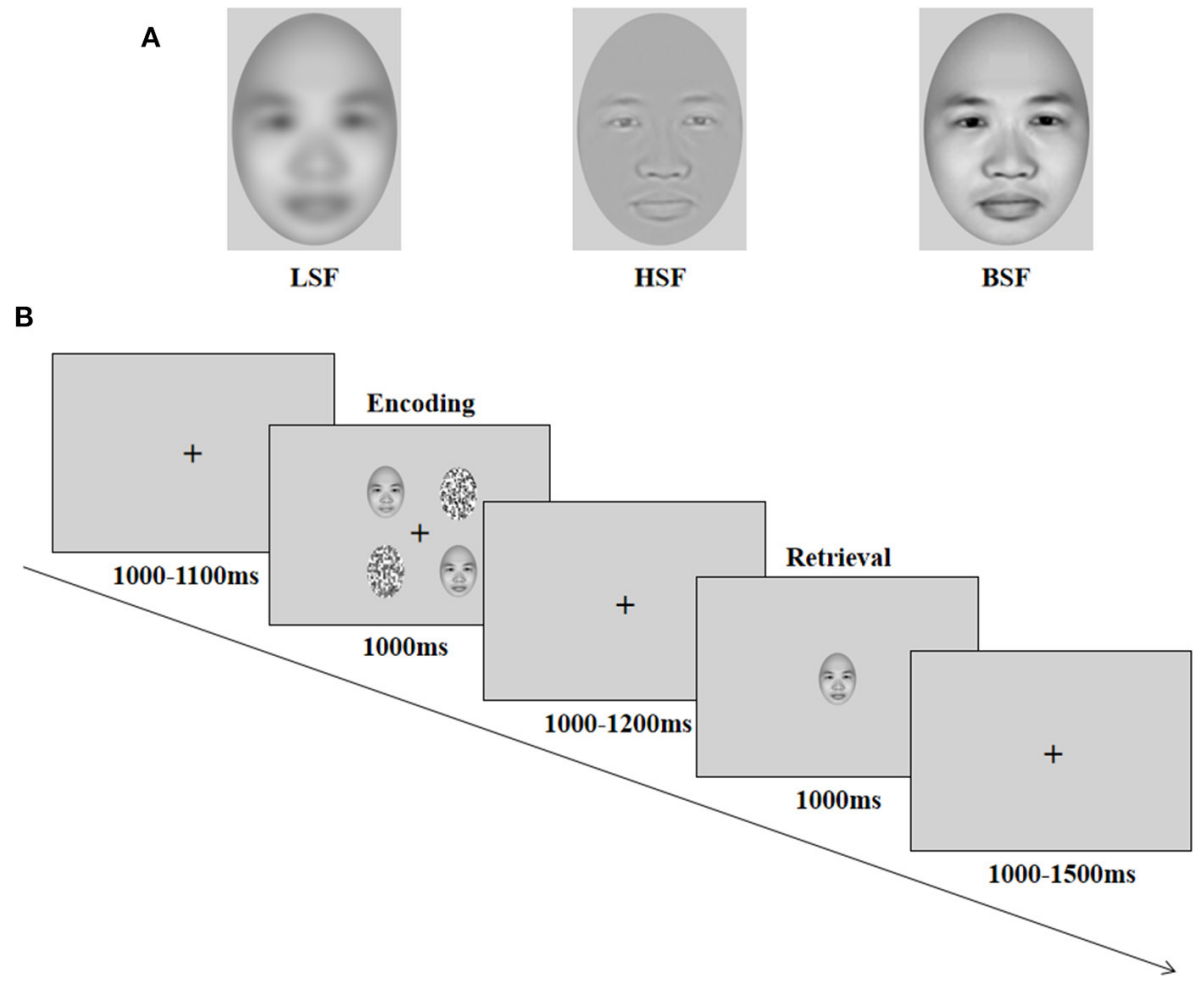
Illustration of face stimuli **(A)** and procedure **(B)**. LSF = low spatial frequency; HSF = high spatial frequency; BSF = broadband spatial frequency. Reproduced with permission from Singular Inversions Inc., available at https://facegen.com.

Each face stimulus was presented without hair or glasses and with a neutral expression, as generated by FaceGen Modeller 3.5 (Toronto, Canada, http://en.softonic.com/) and edited using Adobe Photoshop 7.0 (Adobe Systems, San Jose, CA). According to previous studies (Holmes et al., 2005; Dale and Arnell, 2014), HSF faces were processed in Photoshop using the high-pass filter tool to select only spatial frequencies higher than 6 cycles/degree or 30 cycles/image of visual angle (i.e., a radius of 1.5 pixels); LSF faces were processed in Photoshop using the Gaussian blur tool to select only spatial frequencies lower than 1.3 cycles/degree or 6.5 cycles/image of visual angle (i.e., a radius of 3 pixels); original images with broadband and no spatial frequency filtering were used for BSF faces (Figure 1A). A total of 24 faces were created (8 for each spatial frequency category), with the same grayscale, size, background, luminance, and other physical characteristics. The distractors were the same as those used in previous studies (Wang et al., 2015) and were the scrambled pictures, which were constructed by sectioning each picture into 272 rectangles (in a 7 by 9 matrix) and randomly reassigning the locations of these rectangles, without replacement.

All the stimuli were presented on a 17-in Lenovo monitor (resolution: 1,024 × 768; refresh rate: 60 Hz) using E-Prime 2.0 (Pittsburgh, PA, USA). The viewing distance was 60 cm and the stimulus size was 4° × 5.5° of the visual angle (113 × 156 pixels).

### Procedure

A delayed recognition task was selected for the study, and it allowed separate ERP investigations for encoding and retrieval stages (Morgan et al., 2008). Each trial started with the presentation of a central cross for 1,000–1,100 ms, followed by the encoding display (a 2 × 2 image array) for 1,000 ms. In the encoding display, one, two, or three different face stimuli were presented, corresponding to working memory load 1, load 2, and load 3, respectively, and the remaining stimuli were scrambled distractor images. The four different stimuli were presented randomly on the four corners of the fixation cross, and the eccentricity of the stimuli on the encoding display (measured as the distance between the center of each stimulus and the horizontal or vertical line of the central fixation cross) was 3.8° for stimuli in the four positions (Figure 1B). Participants were instructed to remember the identity of the faces and ignore the scrambled distractors. The stimuli presentation was followed by a 1,000–1,200 ms delay. A test face was then presented on the center of the screen for 1,000 ms (retrieval display), and participants were required to judge whether the test face appeared on the encoding display by pressing the keyboard (50% trials for “yes,” 50% trials for “no”). The intertrial interval was at random and ranged between 1 and 1.5 s.

A 3 (WM load: load 1, load 2, load 3) × 3 (face spatial frequency: low, high, broadband) within-subject design was adopted. Each condition had 120 trials, for a total of 1,080 trials.

### Data Recording and Analysis

EEG data were collected from 64 channels with an EEG recording system produced by the NeuroScan company. Vertical electrooculography (VEOG) was recorded with two electrodes in the upper and lower parts of the left orbital frontal region, and horizontal electrooculography (HEOG) was recorded with two electrodes placed 1.5 cm laterally in both eyes. The reference electrode was located between Cz and CPz. The resistance between all electrodes and the scalp was less than 5 kΩ. The band-pass filter range was 0.01–100 Hz, and the sampling rate was 500 Hz.

EEG data were analyzed using Letswave 7 software (https://letswave.cn; Mouraux and Iannetti, 2008) operating in Matlab R2013b (Mathworks, Natick, MA, USA). At both encoding and retrieval stages, the EEG analysis window was between −200 to 700 ms, and a baseline was acquired 200 ms before the appearance of stimuli. EEG data were band-pass filtered at 0.1–30 Hz, and the average of all electrodes was used as a reference. Eye artifacts were identified by independent components analysis and removed from all EEG electrode traces, and the rejection standard was ± 75 mV. Only trials with correct responses were analyzed for the retrieval stage.

According to previous studies (Wang et al., 2015, 2016, 2020), we analyzed P1 (75–145 ms), N170 (140–200 ms), and P2 components (200–270 ms) acquired from P3/P4, P5/P6, P7/P8, PO3/PO4, PO5/PO6, PO7/PO8, and O1/O2 electrodes. Based on previous studies (Morgan et al., 2008; Bauser et al., 2011), P3b (300–600 ms) was analyzed from PZ, POZ, and OZ electrodes to examine WM load manipulation. Besides, our stimulus number is low, which might make the faces familiar. To test the familiarity effect, the mean amplitude of N250r (230–320 ms) was analyzed from P7/P8, PO7/PO8, and O1/O2 electrodes (Neumann and Schweinberger, 2008). The latency (time from start to peak) and amplitude (baseline to peak) of each component were selected as dependent variables. A 3 (face spatial frequency: BSF, LSF, HSF) × 3 (WM load: load 1, load 2, load 3) × 2 (hemisphere: left, right) repeated-measures ANOVA was performed for P1, N170, P2, and N250r components. A 3 (WM load: load 1, load 2, load 3) × 3 (face spatial frequency: BSF, LSF, HSF) repeated-measures ANOVA was performed on reaction time and accuracy performance and the P3b component. When necessary, *p* values were corrected using the Greenhouse-Geisser method. The Bonferroni correction was applied to account for multiple comparisons and *post-hoc* analyses were performed to understand interaction effects.

## Results

### Behavioral Performance

There was a significant main effect of face spatial frequency on reaction time [*F*_(2, 54)_ = 3.51, *p* = 0.042, η2 *p* = 0.115]; however, *post-hoc* analyses did not detect any differences among the three SFs (LSF: 652 ms, HSF: 661 ms, BSF: 642 ms). A significant main effect of WM load was detected on reaction time [*F*_(2, 54)_ = 25.44, *p* < 0.001, η2 *p* = 0.485], and *post-hoc* analyses determined that the reaction time for load 1 was shorter than for load 2 and load 3 (614 vs. 667 vs. 674 ms, *p* < 0.001). The interaction between face spatial frequency and WM load on reaction time was not significant [*F*_(4, 108)_ = 1.54, *p* = 0.209].

There was a significant main effect of face spatial frequency on accuracy [*F*_(2, 54)_ = 39.13, *p* < 0.001, η2 *p* = 0.592], and *post-hoc* results revealed that accuracy was higher for BSF than for LSF and HSF conditions (79 vs. 74 vs. 71%, *p* < 0.05). The decomposition of a significant main effect of WM load on accuracy [*F*_(2, 54)_ = 321.86, *p* < 0.001, η2 *p* = 0.923] showed that accuracy decreased with increasing WM load (load 1: 88%, load 2: 75%, load 3: 62%, *p* < 0.001). Importantly, the interaction effect of face spatial frequency and WM load on accuracy was significant [*F*_(4, 108)_ = 3.22, *p* = 0.018, η2 *p* = 0.107]. The accuracy for detection of BSF faces was higher than for LSF and HSF faces under load 1 [91 vs. 87 vs. 85%, *F*_(2, 54)_ = 17.73, *p* < 0.001, η2 *p* = 0.396] and load 2 (80 vs. 73 vs. 70%, *F*_(2, 54)_ = 26.25, *p* < 0.001, η2 *p* = 0.493), and it was significantly different among BSF, LSF, and HSF conditions under load 3 (67 vs. 62 vs. 58%, *F*_(2, 54)_ = 34.27, *p* < 0.001, η2 *p* = 0.559). These results indicate that HSF appears to be less important than LSF under increasing WM load.

### ERP Results

**Figure 2:**
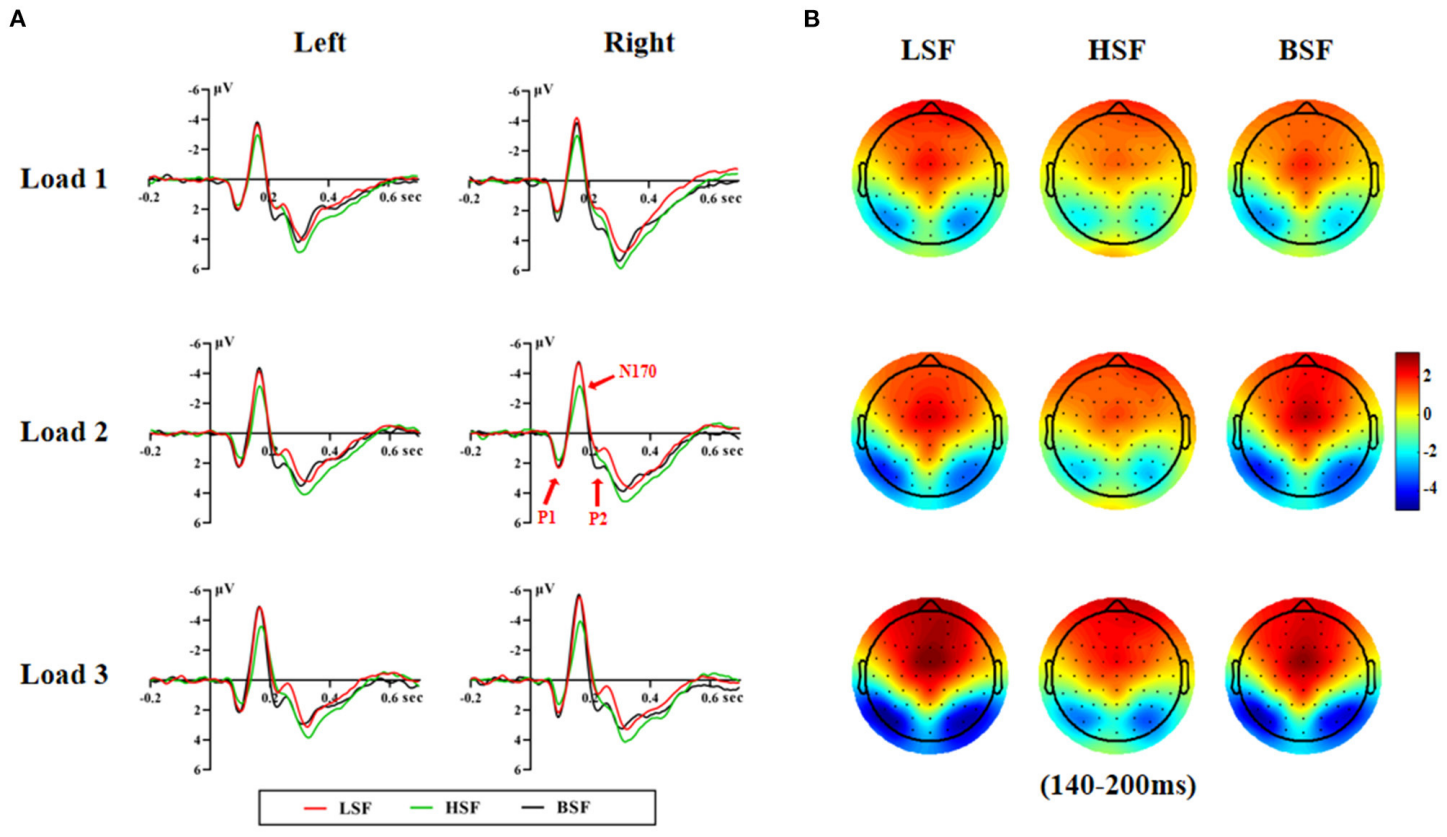
Grand averaged ERP waveforms on the left and right hemispheres **(A)** and the topographic maps **(B)** during the encoding stage.

**Figure 3:**
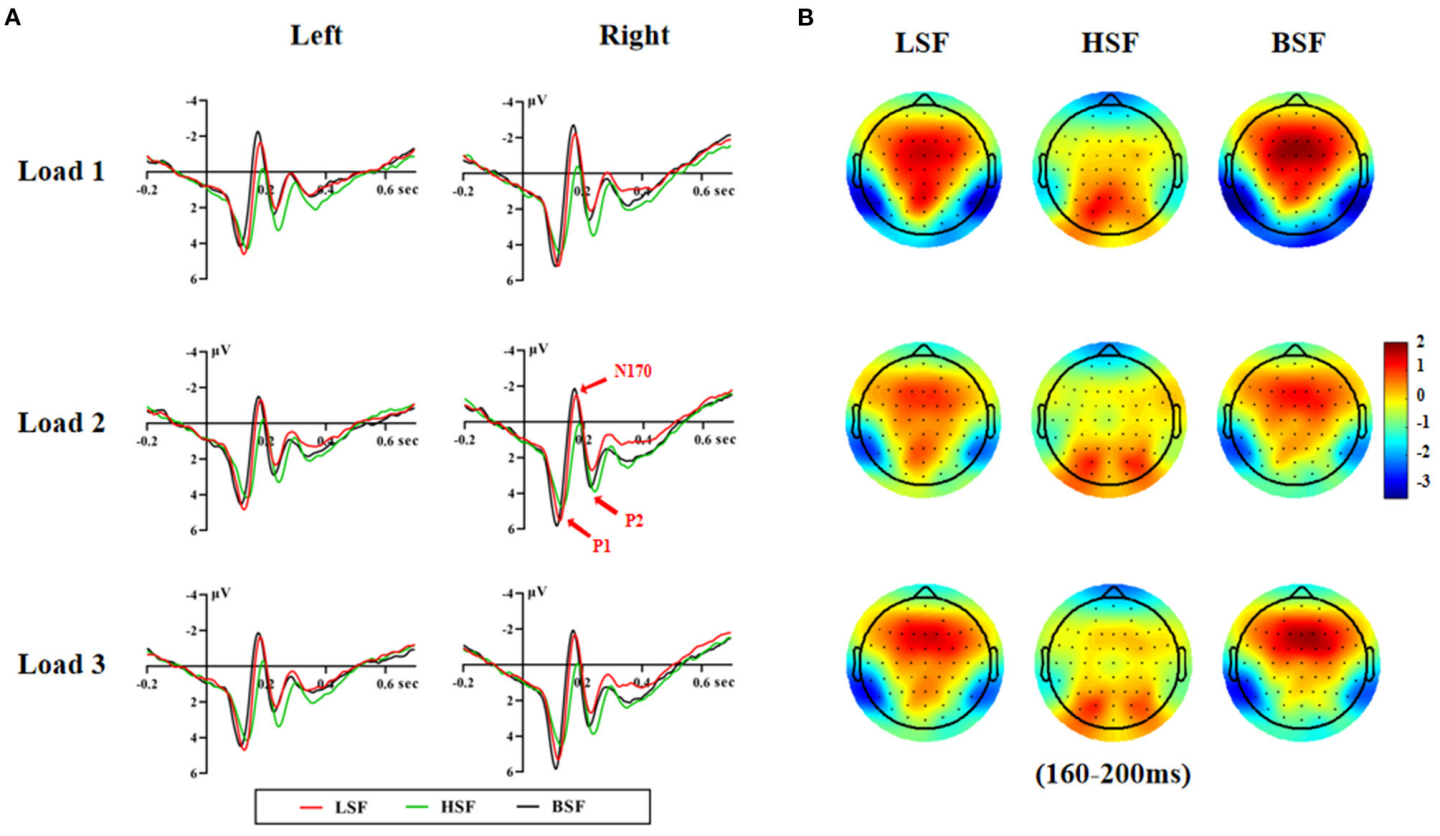
Grand averaged ERP waveforms on the left and right hemispheres **(A)** and the topographic maps **(B)** during the retrieval stage.

Figures 2, 3 show grand-average ERPs elicited by BSF, HSF, and LSF faces during encoding and retrieval stages, recorded from the temporal-occipital cortex. All stimuli evoked the canonical P1, N170, P2, P3b, and N250r components. Table 1 shows the results on P1, N170, and P2 amplitude and latency during the encoding and retrieval stages.

**Table 1:** The amplitude (μV) and latency (ms) values of the P1, N1, and P2 components for low-spatial frequency (LSF) and high-spatial frequency (HSF) face during the encoding and retrieval stages.

	**P1**			**N170**			**P2**		
	**LSF**	**HSF**	***p***	**LSF**	**HSF**	**p**	**LSF**	**HSF**	***p***
Amplitude									
Encoding	3.10 ± 0.29	2.88 ± 0.26	0.161	−5.16 ± 0.66	−4.00 ± 0.64	0.001	2.33 ± 0.77	2.56 ± 0.75	0.678
Retrieval	6.21 ± 0.34	5.80 ± 0.35	0.099	−2.26 ± 0.52	−1.08 ± 0.56	0.001	3.02 ± 0.51	4.34 ± 0.50	0.001
Latency									
Encoding	100 ± 2	101 ± 2	0.459	163 ± 2	164 ± 2	0.614	231 ± 2	235 ± 2	0.029
Retrieval	122 ± 2	125 ± 2	0.061	179 ± 2	183 ± 2	0.120	232 ± 2	240 ± 2	0.001

#### Encoding Stage

##### P1

There was a significant main effect of face spatial frequency on P1 amplitude and latency [amplitude: *F*_(2, 56)_ = 7.30, *p* < 0.01, η2 *p* = 0.207; latency: *F*_(2, 56)_ = 8.30, *p* < 0.001, η2 *p* = 0.229]. BSF faces evoked larger and earlier P1 responses than HSF faces (3.23 vs. 2.88 μV, *p* = 0.005; 97 vs. 101 ms, *p* = 0.001).

##### N170

There were significant main effects of face spatial frequency [*F*_(2, 56)_ = 26.30, *p* < 0.001, η2 *p* = 0.484] and WM load [*F*_(2, 56)_ = 61.00, *p* < 0.001, η2 *p* = 0.685] on N170 amplitude. The N170 amplitude elicited by LSF faces and BSF faces was larger than for HSF faces (−5.16 vs. −5.23 vs. −4.00 μV, *p* < 0.001). Besides, there was a significant interaction between WM load and hemisphere on N170 amplitude [*F*_(2, 56)_ = 4.13, *p* < 0.05, η2 *p* = 0.129]. *Post-hoc* analyses showed that the N170 amplitude elicited by load 3 was larger than that for load 2 and load 1 on both hemispheres [left: −5.07 vs. −4.47 vs. −4.03 μV, *F*_(2, 56)_ = 34.83, *p* < 0.001, η2 *p* = 0.554; right: −5.85 vs. −4.96 vs. −4.41 μV, *F*_(2, 56)_ = 52.06, *p* < 0.001, η2 *p* = 0.650]. These results indicated that N170 amplitude was more sensitive to configural face processing than featural face processing, which was not modulated by WM load.

There were significant main effects of spatial frequency [*F*_(2, 56)_ = 4.19, *p* = 0.033, η2 *p* = 0.130] and WM load [*F*_(2, 56)_ = 16.20, *p* < 0.001, η2 *p* = 0.367] on N170 latency. More importantly, the interaction between spatial frequency and WM load was significant [*F*_(4, 112)_ = 6.78, *p* < 0.001, η2 *p* = 0.195]. The N170 latency for BSF faces (161 ms) was earlier than that for LSF (166 ms) and HSF faces (168 ms) under the load 3 condition [*F*_(2, 56)_ = 7.74, *p* < 0.001, η2 *p* = 0.217]. There were no significant differences in N170 latency among spatial frequencies for the load 1 and load 2 conditions.

##### P2

There were significant main effects of spatial frequency [amplitude: *F*_(2, 56)_ = 20.53, *p* < 0.001, η2 *p* = 0.423; latency: *F*_(2, 56)_ = 7.87, *p* = 0.002, η2 *p* = 0.219] and WM load (amplitude: *F*_[2, 56]_ = 45.52, *p* < 0.001, η2 *p* = 0.619) on P2 amplitude and latency. BSF faces (3.49 μV) elicited a larger P2 than LSF (2.33 μV) and HSF faces (2.56 μV, *p* < 0.001). Load 1 (3.55 μV) elicited a larger P2 than load 2 (2.76 μV) and load 3 (2.07 μV, *p* < 0.001).

There was also a significant interaction effect of spatial frequency and WM load on P2 latency [*F*_(4, 112)_ = 3.93, *p* = 0.008, η2 *p* = 0.123]. Furthermore, the interaction of spatial frequency and WM load and hemisphere was also significant [*F*_(4, 112)_ = 3.09, *p* = 0.023, η2 *p* = 0.099]. *Post-hoc* analysis showed that the P2 latency evoked by HSF faces was later than that for LSF and BSF faces [239 vs. 229 vs. 231 ms, *F*_(2, 56)_ = 14.61, *p* < 0.001, η2 *p* = 0.343] on the right hemisphere for the load 3 condition. On the left hemisphere, the interaction between spatial frequency and WM load was not significant. These results indicated that configural face information was processed earlier than featural face information in the right hemisphere under a higher WM load.

##### P3b

There was a significant main effect of WM load on P3b amplitude [*F*_(2, 56)_ = 31.54, *p* < 0.001, η2 *p* = 0.530], and *post-hoc* analyses showed that the amplitude elicited by load 1 was larger than that for load 2 and load 3 (load 1: 4.51 μV; load 2: 3.79 μV; load 3: 3.79 μV, *p* < 0.001).

##### N250r

Results revealed the significant main effects of WM load [*F*_(2, 56)_ = 50.87, *p* < 0.001, η2 *p* = 0.645] and face spatial frequency [*F*_(2, 56)_ = 18.85, *p* < 0.001, η2 *p* = 0.402] on the N250r amplitude. The N250r amplitude decreased with the increase in WM load (3.25 vs. 2.15 vs. 1.17 μV, *p* < 0.01). LSF faces elicited a lower N250r than HSF faces and BSF faces (1.41 vs. 2.65 vs. 2.50 μV, *p* < 0.01).

#### Retrieval Stage

##### P1

There were significant main effects of face spatial frequency [*F*_(2, 56)_ = 4.83, *p* < 0.05, η2 *p* = 0.147] and WM load [*F*_(2, 56)_ = 3.65, *p* < 0.05, η2 *p* = 0.115] on P1 amplitude. The interaction of spatial frequency and hemisphere on P1 amplitude was significant [*F*_(2, 56)_ = 5.89, *p* < 0.01, η2 *p* = 0.174], which upon decomposition indicated that BSF faces evoked larger P1 amplitudes than HSF faces on the right hemisphere [6.56 vs. 5.80 μV, *F*_(1, 28)_ = 16.06, *p* < 0.001, η2 *p* = 0.364].

There were also significant main effects of spatial frequency [*F*_(2, 56)_ = 56.36, *p* < 0.001, η2 *p* = 0.668] and hemisphere [*F*_(1, 28)_ = 6.68, *p* < 0.05, η2 *p* = 0.193] on P1 latency. P1 latencies elicited by BSF faces were earlier than for LSF and HSF faces (112 vs. 122 vs. 125 ms, *p* < 0.001), and they were also earlier on the right relative to the left hemisphere (118 vs. 122 ms, *p* < 0.05).

##### N170

**Figure 4:**
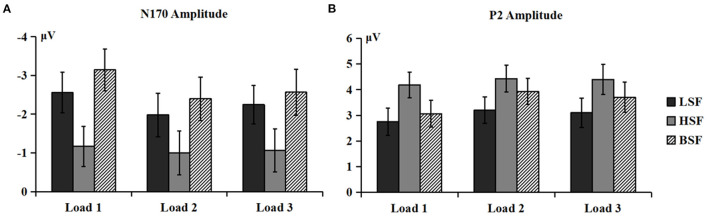
Mean amplitudes of N170 **(A)** and P2 **(B)** components (P3/P4, P5/P6, P7/P8, PO3/PO4, PO5/PO6, PO7/PO8, and O1/O2) elicited by LSF, HSF, and BSF faces under different WM load conditions.

There were significant main effects of face spatial frequency [*F*_(2, 56)_ = 30.36, *p* < 0.001, η2 *p* = 0.520] and WM load [*F*_(2, 56)_ = 7.85, *p* < 0.01, η2 *p* = 0.219] on N170 amplitude. There were also significant interaction effects between WM load and hemisphere [*F*_(2, 56)_ = 7.15, *p* < 0.001, η2 *p* = 0.203] and between spatial frequency and WM load [*F*_(4, 112)_ = 2.85, *p* < 0.05, η2 *p* = 0.092] on N170 amplitude (Figure 4A). *Post-hoc* analysis showed that the N170 amplitudes elicited by HSF faces were lower than those from LSF and BSF faces under the load 1 [−1.17 vs. −2.56 vs. −3.14 μV, *F*_(2, 56)_ = 31.60, *p* < 0.001, η2 *p* = 0.530], load 2 [−1.00 vs. −1.98 vs. −2.40 μV, *F*_(2, 56)_ = 19.76, *p* < 0.001, η2 *p* = 0.414], and load 3 conditions [−1.06 vs. −2.25 vs. −2.57 μV, *F*_(2, 56)_ = 21.28, *p* < 0.001, η2 *p* = 0.432]. These results indicated that N170 was more sensitive to configural face processing than featural face processing, especially under the load 1 condition.

There were significant main effects of face spatial frequency [*F*_(2, 56)_ = 37.24, *p* < 0.001, η2 *p* = 0.571] and hemisphere [*F*_(1, 28)_ = 8.72, *p* < 0.01, η2 *p* = 0.237] on N170 latency. N170 latency was later for LSF and HSF faces than BSF faces (179 vs. 183 vs. 171 ms, *p* < 0.001) and was later in the left hemisphere compared to the right hemisphere (180 vs. 176 ms).

##### P2

There were significant main effects of face spatial frequency [*F*_(2, 56)_ = 14.05, *p* < 0.001, η2 *p* = 0.334] and WM load [*F*_(2, 56)_ = 10.13, *p* < 0.001, η2 *p* = 0.266] on P2 amplitude. The interaction between WM load and hemisphere was significant [*F*_(2, 56)_ = 5.61, *p* =0.015, η2 *p* = 0.167]. The load 1 P2 amplitude was lower than that for load 2 and load 3 [3.42 vs. 4.13 vs. 4.03 μV, *F*_(2, 56)_ = 14.60, *p* < 0.001, η2 *p* = 0.343] in the right hemisphere. Importantly, the interaction of spatial frequency and WM load was also significant [*F*_(4, 112)_ = 2.75, *p* = 0.037, η2 *p* = 0.089; Figure 4B]. HSF faces evoked larger P2 amplitudes than LSF and BSF faces [4.18 vs. 2.76 vs. 3.06 μV, *F*_(2, 56)_ = 15.58, *p* < 0.001, η2 *p* = 0.357] under load 1. Amplitudes were larger for HSF and BSF faces than LSF faces [4.43 vs. 3.93 vs. 3.20 μV, *F*_(2, 56)_ = 10.77, *p* < 0.001, η2 *p* = 0.278] under load 2, with no significant difference between P2 amplitudes from HSF and BSF faces. For load 3, HSF faces gave larger amplitudes than LSF faces [4.40 vs. 3.10 μV, *F*_(1, 28)_ = 24.54, *p* < 0.001, η2 *p* = 0.467], while P2 amplitude from BSF faces did not differ with that from either LSF or HSF faces. These results indicated that P2 was more sensitive to featural face processing than configural face processing, especially under the load 3 condition.

There were significant main effects of face spatial frequency [*F*_(2, 56)_ = 27.90, *p* < 0.001, η2 *p* = 0.499] on P2 latency. There were significant differences among the P2 latencies elicited by BSF, LSF, and HSF faces (229 vs. 232 vs. 240 ms, *p* < 0.05).

##### P3b

Results revealed a significant main effect of WM load [*F*_(2, 56)_ = 6.73, *p* < 0.05, η2 *p* = 0.194] on P3b amplitude. P3b amplitude was lower for load 3 than for load 2 and load 1 (−0.171 vs.0.048 vs.0.339 μV, *p* < 0.05).

##### N250r

There was a significant main effect of WM load [*F*_(2, 56)_ = 17.64, *p* = 0.000, η2 *p* = 0.387], due to load 1 eliciting a more negative N250r amplitude relative to other conditions (−0.648 vs. 0.356 vs. 0.158 μV, *p* < 0.01). In addition, the main effect of face spatial frequency was significant [*F*_(2, 56)_ = 8.58, *p* = 0.002, η2 *p* = 0.235], showing that LSF faces induced a more negative amplitude than HSF faces (−0.60 vs. 0.64 μV, *p* < 0.01).

## Discussion

This study investigated whether and how working memory load impacts coarse-to-fine face processing during WM encoding and retrieval stages. Our findings showed that for the encoding stage, P1 and P2 amplitudes were more sensitive to BSF relative to HSF faces, while N170 amplitude was more sensitive to LSF and BSF relative to HSF faces. P1 and N170 (under the load 3 condition) latencies were earlier for BSF than HSF faces, and P2 latency on the right hemisphere was earlier for LSF and BSF than HSF faces under the load 3 condition. The results indicated that featural face information appears to be less important than configural face information during the WM encoding stage (from N170 to P2 component). For the retrieval stage, P1 in the right hemisphere was more greatly influenced by BSF than HSF faces. N170 amplitude was more sensitive to BSF and LSF faces than HSF faces, especially under the load 1 condition. P2 amplitude was more influenced by HSF faces than LSF faces, especially under the load 3 condition. BSF faces had earlier latency than LSF and HSF faces among P1, N170, and P2. The results indicated that face perception follows a coarse-to-fine sequence during the WM retrieval stage. Specifically, coarse information (i.e., LSF, on N170) was processed first and more rapidly, followed by the processing of fine information (i.e., HSF, on P2). Additionally, P3b amplitude decreased as WM load increased at both the encoding and retrieval stages, which is consistent with previous studies (Morgan et al., 2008) and shows the successful manipulation of WM load. LSF faces induced more negative N250r than HSF faces during both encoding and retrieval stages, which was in line with the idea that familiar faces are processed in a configural manner relative to unfamiliar faces (Ramon and Rossion, 2012).

### The LSF and HSF Face Processing During the Encoding Stage

Previous studies reported that P1 was a robust component that was associated with holistic face processing and was more sensitive to LSF faces (Nakashima et al., 2008; Jeantet et al., 2019), which was in line with the assumption that configural information plays an essential role in early face processing as it could efficiently provide the whole structure of the face (Freire et al., 2000; James et al., 2001; Itier and Taylor, 2002, 2004). Conversely, others reported that P1 was more sensitive to BSF faces (Pourtois et al., 2005; Peters et al., 2013; Craddock et al., 2015). Consistent with these latter findings, our results showed that BSF faces elicited a larger and earlier P1 relative to HSF faces at the encoding stage. BSF faces were from the upright non-filtered faces, which are processed in a more configural way compared with inverted faces (Schwaninger et al., 2003; Kimchi and Amishav, 2010; Cousins et al., 2020). Interestingly, we found that LSF and HSF faces cannot be purely dissociated based on the early P1 component during the encoding stage, which might be reconciled by the assumption that HSF faces also inevitably contain configural information, less than LSF faces but enough to produce an effect similar to LSF faces during early face processing (Halit et al., 2006). The cutoff value of spatial frequency, which varied depending on studies, might be another important factor. LSF values were fewer than 8, 5, 4, or 3 cycles/image, and HSF values were above 35, 30, 24, 22, or 15 cycles/image (Jeantet et al., 2019). Our LSF and HSF faces might include mid-band spatial frequencies (MSF), which are important in face processing (for a review, see Jeantet et al., 2018). Thus, our results suggest that P1 was less sensitive to LSF and HSF faces during the encoding stage.

The impact of spatial frequencies on N170 modulation has been controversial. Our results replicated previous findings that N170 was more negative following the presentation of BSF or LSF faces relative to HSF faces during the encoding stage (Goffaux et al., 2003; Peters et al., 2013; Yao and Zhao, 2019), but are in contrast to other studies reporting a larger N170 in response to HSF faces (Nakashima et al., 2008; Jeantet et al., 2019) or reporting no association between N170 amplitude and face spatial frequency (Holmes et al., 2005). How can these discrepancies be reconciled? One answer may stem from selective attention to spatial location. Selective attention to objects in the periphery promotes LSF processing, and attention to foveal location promotes HSF processing (Shulman and Wilson, 1987; Carrasco et al., 2006). In this study, face stimuli were randomly presented in the four corners of the fixation cross during the encoding stage, directing attention to the periphery, hence the N170 amplitude was greater for LSF faces relative to HSF faces. Thus, the current N170 reflected the configural processing during the encoding stage.

Although many studies found that P2 is more sensitive to LSF pictures than HSF pictures (Mathes and Fahle, 2007; De Cesarei et al., 2013; Yang and Chan, 2015), P2 amplitude is also augmented by visual stimuli with broadband information (Hansen et al., 2011). In this study, P2 amplitude was more sensitive to BSF faces than to LSF and HSF faces during the encoding stage. According to previous research (Craddock et al., 2015), this might be related to the fact that BSF faces were upright non-filtered faces, which had higher spectral power and could drive stronger responses compared to faces with lower or higher spectral power. In addition, BSF faces contain not only configural and featural information but also MSF information (Halit et al., 2006). Previous research found that MSF information plays an important role in face processing (Collin et al., 2012; Jeantet et al., 2018) as it is intermixed with LSF and HSF information and can be processed in a similar way to the non-filtered BSF faces (Jeantet et al., 2019). Moreover, Parker and Costen (1999) demonstrated that recognition was more accurate and rapid for MSF faces than for LSF or HSF faces, which was also supported by later ERP research (Collin et al., 2012). These combined results led us to postulate that it might be the MSF information that made P2 highly sensitive to BSF faces during the encoding stage. Thus, our finding indicated that LSF and HSF faces cannot be dissociated from P2 amplitude during the encoding stages. But, the P2 latency showed that LSF faces were processed earlier than HSF faces.

In sum, for the encoding stage, LSF and HSF faces cannot be dissociated on the P1 component but can be dissociated on the N170 and P2 components, which were more sensitive to LSF faces than to HSF faces. The result is in line with the hypothesis that the N170 is a marker for face structural encoding and is linked to configural face processing (Eimer, 2011).

### The Time Course of LSF and HSF Face Processing During the Retrieval Stage

Consistent with our results at the encoding stage, the results at the retrieval stage showed that P1 was less sensitive to LSF and HSF faces and that N170 was more sensitive to LSF faces than HSF faces. However, the P2 was more positive for HSF faces relative to BSF and LSF faces during the retrieval stage, suggesting that P2 was more sensitive to face featural information. Although the sensitivity of P2 to featural processing has rarely been observed, several previous studies reported that faces with featural modifications elicited a larger P2 amplitude than faces with second-order relational modifications (Wang et al., 2016; Wang and Fu, 2018). Moreover, using Chinese and Western participants, Wang et al. (2020) found that P2 in Chinese participants was more sensitive to own-race faces with featural modifications. The reverse result was observed for Western participants, suggesting that cultural variation might account for this discrepancy.

It is worth emphasizing that the P2-enhancing effect of HSF faces was only observed at the retrieval stage and not at the encoding stage. It might be that faces in the encoding phase were processed in a parallel way not relying on in-depth visual analysis (cortical visual feedback; Mercure et al., 2008). Therefore, coarse facial information was more important during encoding, while during memory retrieval finer featural processing was adopted to match the encoded faces to the target at late stages (Bauser et al., 2011). Moreover, P2 could reflect the comparison between experimental face stimulus features and mental templates for task-related features (Potts, 2004); hence the facial features were of particular importance during the retrieval stage and evoked larger P2 waveforms. Taken together, only P2 reflected that encoding and retrieval stages were dissociated for face spatial frequencies.

In sum, for the retrieval stage, N170 was also more sensitive to LSF faces than to HSF faces, whereas P2 was more sensitive to HSF faces than to LSF faces. The processing priority of LSF faces during the retrieval stage is in line with previous neuronal findings that information processed in the LSF-sensitive magnocellular pathway has a faster cortical arrival than information processed in the HSF-sensitive parvocellular pathway (Laycock et al., 2007).

### The Role of WM Load in Face SF Processing

This study found that WM load modulated face SF processing, which was reflected in the behavioral results and ERP responses. Consistent with previous studies (Morgan et al., 2008; Bauser et al., 2011), the P3b amplitude decreased as WM load increased during both the encoding and retrieval stages. Moreover, at the retrieval stage, the N170 amplitude evoked by LSF and BSF faces did not differ but was larger than that for HSF faces, especially under the load 1 condition. These results indicated that BSF faces tended to be processed in a configural manner under a lower WM load, which agrees with previous findings that N170 reflects first-order relational processing (Maurer et al., 2002). However, our behavioral results showed higher accuracy in processing LSF faces relative to HSF faces under the high WM load condition, which might be related to the limited WM capacity for faces. Previous studies showed that the maintenance capacity for faces is approximately two (Towler et al., 2016), which might not have allowed participants to process the details and features of faces in the higher WM load conditions (load 3).

However, compared with the N170 component, we found a different result for the P2 component during WM retrieval. Specifically, the P2 amplitude evoked by HSF faces was larger than that for LSF faces, especially under the load 3 condition. Besides, BSF faces tended to be processed in a configural manner under the load 1 condition, as there was no difference between BSF and LSF faces. Conversely, they tended to be processed in a featural manner under the load 2 condition, as there was no P2 difference between BSF and HSF faces. BSF faces tended to be processed in both configural and featural manners under the load 3 condition, as there were no differences in P2 evoked by faces in any SF category. These results cannot be reconciled with the proposition from Morgan et al. (2008) that resources are limited during the retrieval stage by the increasing number of faces during the encoding stage. According to Morgan et al. (2008), attentional resources are sufficient under a low WM load, and fine processing of facial features should be adopted; under a high WM load, attentional resources are insufficient, and efficient global/configural processing should be adopted. However, we found the opposite results, and the mechanisms underlying this discrepancy need to be further investigated.

Notably, we also found a hemispheric advantage for N170 during the encoding and retrieval stages. In agreement with previous results (Rossion et al., 2003; Scott and Nelson, 2006), N170 was larger in the right hemisphere than in the left hemisphere during the encoding stage. Moreover, relative to HSF faces, the LSF faces elicited an earlier P2 under the load 3 condition during the encoding stage on the right hemisphere. This is in line with previous studies showing that configural processing occurs strongly in the right hemisphere (Scott and Nelson, 2006; Calvo and Beltran, 2014; Wang et al., 2016; Worley and Boles, 2016).

### Limitation

Our results might be confined to the familiar faces as the low stimuli number is likely to make the faces familiar in this study. We analyzed N250r, which is more sensitive to familiar faces than unfamiliar faces, to test the familiarity effect. Compared with HSF faces, LSF faces elicited more negative N250r in the present. This result provides evidence that familiar faces are more sensitive to configural information, even though there is an argument against this idea (Burton et al., 2015). Additionally, our data showed that the N250r amplitude decreased as the WM load increased during the encoding (from load 1 to load 2) and retrieval stages (from load 1 to load 3). The results were consistent with previous studies (Morgan et al., 2008), which employed six male faces. Further work is required to minimize the effect of familiarity. Another factor that cannot be neglected is the cutoff value of face spatial frequency. As we mentioned above, this value varied depending on studies, which might lead to different results. Compared with previous studies, our LSF (below 6.5 cycles/image) and HSF (above 30 cycles/image) faces might include the mid-band spatial frequencies [6.73–32 cycles/image in Collin et al. (2012); 5–15 cycles/image in Hsiao et al. (2005)]. Thus, this factor should be taken into consideration when comparing different studies.

## Conclusion

This study indicated that featural face information appears to be less important than configural face information during the working memory encoding stage from 140 to 270 ms, whereas face processing follows a coarse-to-fine manner during the retrieval stage as LSF faces (N170) are processed earlier than HSF faces (P2). Furthermore, the working memory load has an impact on the time course of face processing during the retrieval stage rather than the encoding stage. The results were consistent with face processing models that suggest facial recognition seems to be based on distinct types of processing. This study further shows the dissociation of face processing manner during the encoding and retrieval stages.

## Data Availability Statement

The raw data supporting the conclusions of this article will be made available by the authors, without undue reservation.

## Ethics Statement

The studies involving human participants were reviewed and approved by the local Institutional Review Board of the School of Psychology, Shandong Normal University. The patients/participants provided their written informed consent to participate in this study.

## Author Contributions

HW designed the research. HZ and EC obtained the data. AW, EC, and CB analyzed the data. AW and HW wrote the manuscript. All authors contributed to the article and approved the submitted version.

## Funding

This study was supported by the National Natural Science Foundation of China [Grant Numbers 32171042 and 31700940] for HW.

## Conflict of Interest

The authors declare that the research was conducted in the absence of any commercial or financial relationships that could be construed as a potential conflict of interest.

## Publisher's Note

All claims expressed in this article are solely those of the authors and do not necessarily represent those of their affiliated organizations, or those of the publisher, the editors and the reviewers. Any product that may be evaluated in this article, or claim that may be made by its manufacturer, is not guaranteed or endorsed by the publisher.
